# Completeness and accuracy of national cancer and death registration for outcome ascertainment in trials—an ovarian cancer exemplar

**DOI:** 10.1186/s13063-020-04968-x

**Published:** 2021-01-25

**Authors:** Jatinderpal K. Kalsi, Andy Ryan, Aleksandra Gentry-Maharaj, Danielle Margolin-Crump, Naveena Singh, Matthew Burnell, Elizabeth Benjamin, Sophia Apostolidou, Mariam Habib, Susan Massingham, Chloe Karpinskyj, Robert Woolas, Martin Widschwendter, Lesley Fallowfield, Stuart Campbell, Steven Skates, Alistair McGuire, Max Parmar, Ian Jacobs, Usha Menon

**Affiliations:** 1 Department of Women’s Cancer, Institute for Women’s Health, University College London, London, WC1E 6AU UK; 2 MRC Clinical Trials Unit at UCL, Institute of Clinical Trials & Methodology, University College London, 90 High Holborn, 2nd Floor, London, WC1V 6LJ UK; 3 Department of Cellular Pathology, Barts Health NHS Trust, London, E1 2ES UK; 4 HCA International, Pathology Laboratories, London, WC1E 6JA UK; 5 Imperial Clinical Trials Unit, Imperial College London, London, W12 7RH UK; 6 Department of Gynaecological Oncology, Queen Alexandra Hospital, Portsmouth, PO6 3LY UK; 7 Sussex Health Outcomes Research and Education in Cancer (SHORE-C), Brighton and Sussex Medical School, University of Sussex, Brighton, BN1 9RX UK; 8 Create Health, London, EC2V 6ET UK; 9 Massachusetts General Hospital and Harvard Medical School, Boston, MA 02115 USA; 10 London School of Economics, London, WC2A 2AE UK; 11 University of New South Wales, Sydney, NSW 2052 Australia

**Keywords:** Outcomes review, Adjudication, Randomised controlled trial, Ovarian cancer, Screening, UKCTOCS, Registry

## Abstract

**Background:**

There is a trend to increasing use of routinely collected health data to ascertain outcome measures in trials. We report on the completeness and accuracy of national ovarian cancer and death registration in the United Kingdom Collaborative Trial of Ovarian Cancer Screening (UKCTOCS).

**Methods:**

Of the 202,638 participants, 202,632 were successfully linked and followed through national cancer and death registries of Northern Ireland, Wales and England. Women with registrations of any of 19 pre-defined ICD-10 codes suggestive of tubo-ovarian cancer or notification of ovarian/tubal/peritoneal cancer from hospital episode statistics or trial sites were identified. Copies of hospital and primary care notes were retrieved and reviewed by an independent outcomes review committee. National registration of site and cause of death as ovarian/tubal/peritoneal cancer (C56/C57/C48) obtained up to 3 months after trial censorship was compared to that assigned by outcomes review (reference standard).

**Results:**

Outcome review was undertaken in 3110 women on whom notification was received between 2001 and 2014. Ovarian cancer was confirmed in 1324 of whom 1125 had a relevant cancer registration. Sensitivity and specificity of ovarian/tubal/peritoneal cancer registration were 85.0% (1125/1324; 95% CI 83.7–86.2%) and 94.0% (1679/1786; 95% CI 93.2–94.8%), respectively. Of 2041 death registrations reviewed, 681 were confirmed to have a tubo-ovarian cancer of whom 605 had relevant death registration. Sensitivity and specificity were 88.8% (605/681; 95% CI 86.4–91.2%) and 96.7% (1482/1533, 95% CI 95.8–97.6%), respectively. When multiple electronic health record sources were considered, sensitivity for cancer site increased to 91.1% (1206/1324, 95% CI 89.4–92.5%) and for cause of death 94.0% (640/681, 95% CI 91.9–95.5%).

Of 1232 with cancer registration, 8.7% (107/1232) were wrongly designated as ovarian/tubal/peritoneal cancers by the registry and 4.0% (47/1172) of confirmed tubo-ovarian cancers were mis-registered. In 656 with death registrations, 7.8% (51/656) were wrongly assigned as due to ovarian/tubal/peritoneal cancers while 6.2% (40/645) of confirmed tubo-ovarian cancer deaths were mis-registered.

**Conclusion:**

Follow-up of trial participants for tubo-ovarian cancer using national registry data will result in incomplete ascertainment, particularly of the site due in part to the latency of registration. This can be reduced by using other routinely collected data such as hospital episode statistics. Central adjudication by experts though resource intensive adds value by improving the accuracy of diagnoses.

**Trial registration:**

ISRCTN: ISRCTN22488978. Registered on 6 April 2000

## Introduction

Currently, most trials use multiple sources for outcome ascertainment such as case record forms, patient questionnaires and electronic national health datasets. The latter includes cancer and death registrations, hospital administrative records (e.g. hospital episode statistics in the UK), national audit data and specialised health datasets. This is often followed by central adjudication to ensure consistency and minimise bias [1]. Central review requires medical records to be retrieved, checked for completeness, redacted with regard to any reference to randomisation group and patient identifiers and the collated information reviewed by a specialist committee. Given the expensive and time-consuming nature of this process, there is a clear need to evaluate alternative strategies.

There has been an explosion in the use of electronic national datasets for clinical research. As the data quality of such resources improves and access processes for research become more streamlined, this is likely to be a less resource-intensive and less costly option for outcome ascertainment in trials. The use of cancer and death registration alone is especially relevant to large randomised controlled trials of cancer screening where cancer site and disease-specific cause of death (CoD) are primary outcome measures. Data from the US National Lung Screening Trial (NLST) suggests that using death certificate CoD alone (18%; 95% CI 4.2–25.0) would not have impacted on the published (20%; 95% CI 6.7–26.7) lung cancer mortality reduction where the process included central adjudication [2]. This was also noted in the Finnish prostate cancer screening trial [3]. However, other trials have raised concerns about the completeness and accuracy of such data [4]. In the Health Insurance Plan of New York breast screening trial [4], there was no screening benefit based on death certificates alone whereas analysis performed using adjudicated data showed a significant effect. This raises the need for further investigation before universal adoption.

In the United Kingdom Collaborative Trial of Ovarian Cancer Screening (UKCTOCS) [5, 6], follow-up relies on multiple data sources including national electronic health-related datasets with expert adjudication (by outcomes review committee) being used for confirmation of ovarian cancer diagnosis and CoD. This was felt necessary as ovarian cancer often presents late with widely disseminated disease, heightening the possibility of disease misclassification. In addition, decreased investigation in older women, accident and emergency presentation and mortality within the first month of presentation could contribute to ovarian cancer being reported as a malignant neoplasm of unknown origin (ICD-10 code C80) [7]. The trial data provides an opportunity to determine the completeness and accuracy of national ovarian cancer and death registration in England, Wales and Northern Ireland using outcomes review as the reference standard.

## Methods

Between April 2001 and September 2005, 202,638 postmenopausal women aged 50–74 from the general population were recruited. They were randomised to annual screening by transvaginal ultrasound (50,639) or a multimodal strategy using serum CA125 interpreted by the Risk of Ovarian Cancer Algorithm (ROCA), followed by ultrasound as a second-line test (50,640) or control (no intervention; 101,359) [5, 6]. The full trial protocol is accessible at https://www.ctu.mrc.ac.uk/studies/all-studies/u/ukctocs/. Trial registration is ISRCTN22488978. This analysis is based on data collected prospectively up to the end of the initial follow-up phase of UKCTOCS (2001–2014).

Multiple sources were used for the follow-up of trial participants (Table 1) [6]. Women who provided written consent for follow-up through national registries were flagged using their NHS number, date of birth and address for cancer and/or death registrations with NHS Digital (England and Wales) and the Northern Ireland Cancer Registry and Business Services Organisation Health and Social Care Northern Ireland (BSO Northern Ireland), respectively. Censorship data for the initial analysis of trial data was 31 December 2014 [6]. The last notification prior to data freeze for trial outcome analysis was received for both cancer and deaths for England and Wales on 25 March 2015 (NHS Digital), deaths for Northern Ireland on 9 April 2015 (BSO, Northern Ireland) and cancers for Northern Ireland on 15 April 2015 (Northern Ireland Cancer Registry). For women resident in England, data was also available for 2001–2012 from Hospital Episodes Statistics (HES) and up to 2014 from the National Cancer Intelligence Network. Additionally, all women were sent two follow-up postal questionnaires, 3–5 years after randomisation and in 2014. The UKCTOCS coordinating centre also received direct notification from the 13 trial centres and trial participants or their relatives.

**Table 1:** Data sources used for cancer and death notification in UKCTOCS

Data sources for cancer and death notification	
National electronic health-related dataset sources	
Cancer registry	
Death registry	
Hospital Episode Statistics (for women resident in England 2001–2012)	
National Cancer Intelligence Network (NCIN) dataset	
Postal follow-up questionnaires	
Direct communication from participants or their relatives	
Information on trial case record forms/trial teams	

### Outcomes review committee

An outcomes review committee (ORC) was set up to confirm ovarian cancer diagnosis and death. The committee consisted of two pathologists with specialist interest in gynaecological cancer and two gynaecological oncologists. The ORC members were independent of trial conduct and blinded to the randomisation group.

### Identification of cases and collation of evidence

Cases were identified for OR based on a cancer/death registration with any one of 19 preselected International Classifications of Disease (10th Revision, ICD-10) codes (WHO 2003) [7] (Supplementary Table 1, see Additional file 1) or a notification of possible ovarian cancer through the other sources listed above. The preselected ICD-10 codes were defined at trial initiation as those potentially associated with an underlying ovarian cancer [6]. Cases with an ICD-10 C80 (*n* = 936) death registration and a cancer registration of a non-ovarian malignancy were reviewed by a designated gynaecological oncologist and excluded from the below process.

Dedicated coordinating centre personnel initiated the collection of pertinent documents from National Health Service (NHS) Trusts, general practices, local health authorities, private hospitals and hospices. These included copies of medical records, histology, cytology reports, operative notes, diagnostic imaging reports, hospital letters and discharge summaries, multidisciplinary meeting summaries, chemotherapy/radiotherapy notes and autopsy reports. Procurement of the histology report was obligatory in cases where pathological examination had been undertaken. The documents were organised in chronological order and any direct or indirect mention of the randomisation group redacted, prior to submission to ORC. The minimum dataset required for review comprised three separate documents which could include, in addition to those listed above, death certificates and cancer registrations.

### Outcomes review

**Fig. 1:**
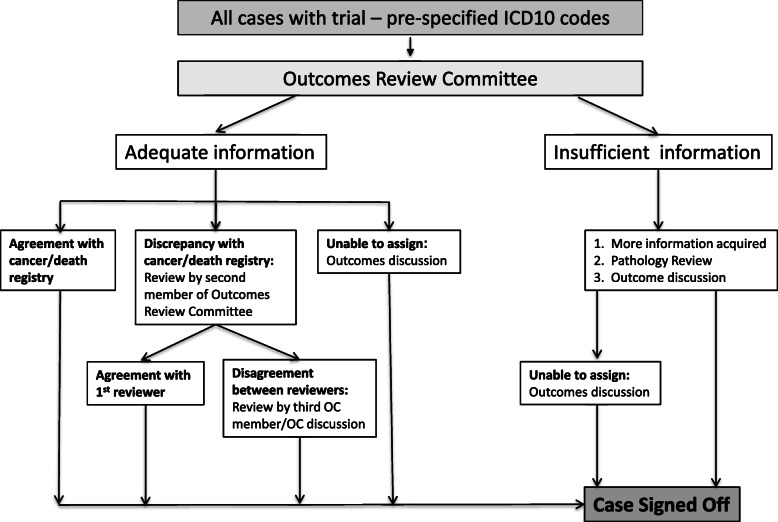
Outcomes review process

Figure 1 shows a schematic for outcomes review. For all cases, a cancer review form (Supplementary Figure 1, see Additional file 2), and where applicable a death review form (Supplementary Figure 2, see Additional file 3), was completed. If the reviewer deemed the documentation adequate, then a cancer diagnosis or CoD was assigned. An algorithm with detailed rules for site allocation (Supplementary Figure 3, see Additional file 4) ensured robust, reproducible and transparent assignment in various clinical scenarios. In all cases where there was a discrepancy regarding ovarian cancer classification between the reviewer and cancer or death registry, the case was forwarded to a second ORC member for an independent review. If both reviewers were in agreement, the assigned diagnosis/CoD was accepted. If there was disagreement, a third ORC member independently reviewed the case and a consensus decision was made based on the majority view. If a reviewer was unable to make a definitive diagnosis, the reviewer could request either further information or review of pathology slides, or a case discussion at an ORC meeting.

Outcomes review initially used the WHO 2003 [7] rules for classifying cases into tubo-ovarian or primary peritoneal. More recently, all the peritoneal cancers were reviewed and reclassified as tubo-ovarian cancers based on WHO revised 2014 criteria [8]. This states that peritoneal cancers can only be diagnosed when there is no demonstrable gross or microscopic ovarian or tubal involvement [9].

### Analysis

**Fig. 2:**
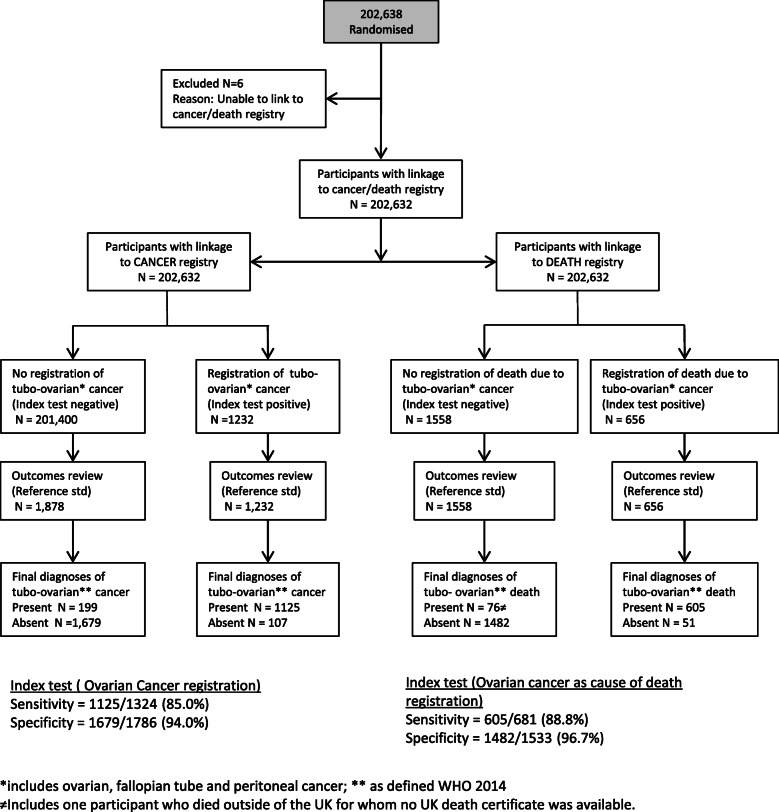
Evaluation of national registrations of tubo-ovarian (includes ovarian, fallopian tube and peritoneal cancer) cancer/death compared to the reference standard (identification through multiple sources, hospital notes retrieval and independent outcomes review)

All trial participants were included in this analysis (Fig. 2). Sensitivities and specificities of cancer/death registration of ovarian cancer obtained up to 3 months postcensorship date were calculated, using central adjudication as the reference standard. We also calculated the sensitivity if all available national electronic health-related dataset sources (Table 1) were used. The definitions for sensitivity and specificity were as defined by the STARD 2015 guidelines (http://www.equator-network.org/reporting-guidelines/stard).

In addition, in all cases with a cancer registration and/or death certification, overall agreement of disease site and CoD between cancer and death registries and OR was assessed using the Cohen’s kappa (*κ*) statistic [10], with confidence intervals calculated using 1000 bootstrap samples. Zero weighting was applied to all off-diagonal cell entries.

We also considered the false-negative rate (FNR = 1-sensitivity) of cancer registration and death certification over time, with ORC-confirmed diagnosis and CoD as the gold standard and summarising with a test for proportional trend (Stata package ptrend). This test takes a standard *χ*^2^ test of cancer registration (or death certification) versus year, on *k* = (row-1) × (column-1) degrees of freedom (df), and partitions the *χ*^2^ value into a linear component (trend test) on 1 df and a ‘departure’ from linearity component on *k*-1 df. We excluded the year 2014 from the cancer registration analysis, as during exploratory analysis there was a large number of missing registrations.

### Funding

The current analysis was funded by a peer-reviewed NIHR HTA grant (16/46/01) and The Eve Appeal. The funders were not involved in the analyses nor in the writing of this report.

## Results

Baseline characteristics of the trial population have been previously described [11]. Of the 202,638 postmenopausal women, 202,632 (99.9%) women were electronically flagged for cancer and death registrations in the relevant national registries of England, Wales and Northern Ireland (Fig. 2).

### Performance characteristics of cancer and death registration for tubo-ovarian cancer

**Fig. 3:**
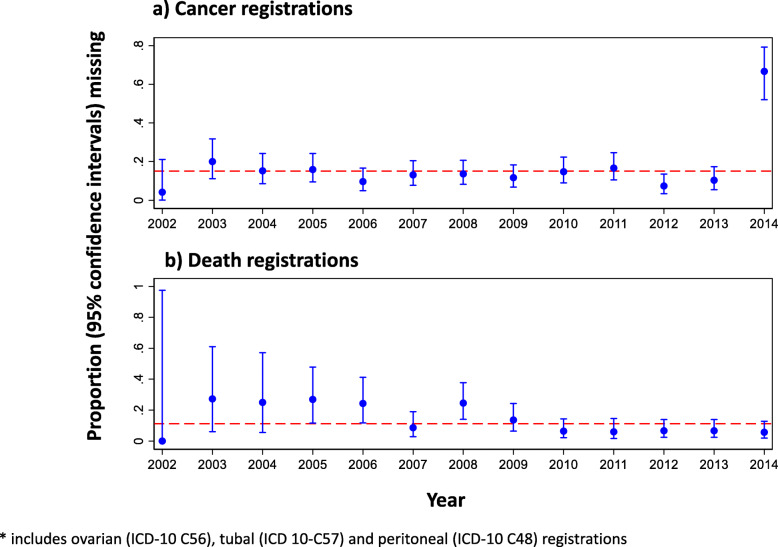
Proportion of missing registrations (includes ovarian (ICD-10 C56), tubal (ICD 10-C57) and peritoneal (ICD-10 C48) registrations) of tubo-ovarian cancers by year of diagnosis

Our algorithm identified 3110 women requiring outcomes review for tubo-ovarian cancer diagnosis. The ORC confirmed 1324 as having tubo-ovarian cancer. Of them, 1125 women had an ovarian, tubal or primary peritoneal cancer registration. Overall sensitivity and specificity of ovarian, tubal or primary peritoneal cancer registration within 3 months of censorship date were 85.0% (1125/1324; 95% CI 83.7–86.2%) and 94.0% (1679/1786; 95% CI 93.2–94.8%), respectively (Fig. 2). In an additional 81 of the 1324 women, we received an ovarian, tubal or primary peritoneal cancer notification from other national datasets (death registration 51, HES 27, NCIN 3). If all available national electronic health-related datasets (Table 1) were considered, sensitivity would be 91.1% (1206/1324; 95% CI 89.4–92.5%). Figure 3a and Supplementary Table 2 (see Additional file 2) show the proportion of missing cancer registrations over time. Excluding the last year (2014), there was no statistical evidence of a trend in the proportion of missing cancer registrations (*p* = 0.261) or any other form of temporal variability (*p* = 0.549).

Our algorithm identified 2214 women who died and required outcomes review for CoD. Only one of these women did not have a death registration as she had died outside the UK. The ORC confirmed 681 as having died due to tubo-ovarian cancer. Of them, 605 women had an ovarian, tubal or primary peritoneal cancer CoD registration (Table 2). Overall sensitivity and specificity of ovarian, tubal or primary peritoneal cancer death registration within 3 months of the censorship date were 88.8% (605/681; 95% CI 86.4–91.2%) and 96.7% (1482/1533; 95% CI 95.8–97.6%), respectively (Fig. 2). In the remaining 76 women, 29 women had an ovarian, tubal or primary peritoneal cancer registration, six had an ovarian cancer HES record. If all available national electronic health-related datasets (Table 1) were considered, sensitivity would be 94.0% (640/681; 95% CI 91.9–95.5%). The false-negative rates by year are presented in Fig. 3b (Supplementary Table 2, see Additional file 5). We found strong evidence of a negative linear trend in the proportion of missing death certificates (*p* = 0.00002) but no further departure from that linearity (*p* = 0.474).

**Table 2:** National cancer and death registrations versus outcomes review confirmation of tubo-ovarian cancer (a) diagnosis and (b) cause of death

Registry assigned	Outcomes review confirmed (reference standard)				Totals
	Tubo-ovarian^a^ cancer (ICD-10 C56 /C57.0)	Malignant neoplasm of unknown origin (C80)	No cancer	Other primary cancer	
**a. Diagnosis (primary cancer site)**					
**Ovarian/tubal or primary peritoneal cancer (ICD-10 C56 /C57/C48)**	1125	33	26	48^b^	**1232**
**Malignant neoplasm of unknown origin (C80)**	9	91	7	48	**155**
**Benign and ovarian neoplasms of uncertain behaviour (ICD-10 D39.1)**	36	0	25	4	**65**
**Other primary cancers**	2	2	0	18	**22**
**Total with registry and outcomes review**	**1172**	**126**	**58**	**118**	**1474**
**b. Cause of death**					
**Ovarian/tubal or primary peritoneal cancer (ICD-10 C56 /C57/C48)**	605	26	6	19^c^	656
**Malignant neoplasm of unknown origin (C80)**	27	272	10	117	426
**Benign and ovarian neoplasms of uncertain behaviour (ICD-10 D39.1)**	2	0	4	0	6
**Other primary cancer**	11	58	2	882	953
**Total**	**645**	**356**	**22**	**1018**	**2041**

^a^WHO 2014, includes cancers previously classified as primary peritoneal cancer

^b^endometrium 20; leiomyosarcoma broad ligament 4; cervix 1; appendix 3; cecum 1; small bowel 2; bile duct 1; breast 2; colon 5; GI stromal 1; lung 1; NHL 3; pancreas 1; skin 1; small lymphocytic lymphoma 1; vulva 1

^c^endometrium (10), colon (3), and one each due to breast cancer, peritoneal mesothelioma, kidney cancer, T cell non-Hodgkin’s lymphoma, lung cancer and oral squamous cell carcinoma

### Agreement of registry- and outcome review-assigned tubo-ovarian cancer diagnosis

There was a relevant ICD-10 cancer registration in 1474 of the 3110 women who underwent outcomes review for potential tubo-ovarian cancer (Table 2). One hundred seven (8.7%; 107/1232) of 1232 cases registered as ovarian, tubal or primary peritoneal cancer were reclassified on outcome review as malignant neoplasm of unknown origin (2.7%; 33/1232), no cancer (2.1%; 26/1232) and other primary cancer (3.9%; 48/1232). Over one third were endometrial (41.6%; 20/48). About one fourth (27.1%; 13/48) of the other primary cancers were metastatic to the ovary.

In addition, 47 (4.0%; 47/1172) of the 1172 women confirmed as having tubo-ovarian cancer by outcomes review would have been missed (Table 2). Almost three fourths of these women were registered as neoplasms of the ovary of uncertain behaviour (ICD-10 D39.1). The majority of these were reclassified on outcomes review as borderline epithelial or non-epithelial ovarian cancer (ICD-10 C56.0). Majority (80.3%; 10/12) of the missed invasive epithelial cancers were at an advanced stage (Table 3). The overall agreement (85.4%; 1259/1474) between the disease site ascribed by the cancer registries and those designated by the ORC was moderate (Cohen’s kappa (*κ*) 0.55; 95% CI 0.5–0.6).

**Table 3:** Characteristics of additional tubo-ovarian cancer (a) cases and (b) deaths identified through adjudication that did not have a national cancer registration

Registry assigned	Designation Following Outcomes Review			
	Number	Histology	Morphology	Stage
**(a) Diagnosis (n = 47)**				
**Neoplasm of uncertain behavior of ovary (ICD-10 D39.1)**	27	Borderline	1 Brenner	1 Stage I
			3 Endometrioid	3 Stage 1a
			9 Mucinous	7 Stage 1a, 2 Stage 1c
			14 Serous	9 Stage 1a, 3 Stage 1c, 1 Stage 111a, 1 Stage 111b
	8	Non-epithelial	7 Granulosa cell	4 Stage Ia, 2 Stage 1c, 1 Stage IIb
			1 Carcinoid	1 Stage Ia
	1	Invasive epithelial	1 Carcinosarcoma	1 Stage IIIc
**Malignant neoplasm without specification of site (ICD-10 C80)**	9	Invasive epithelial	2 Carcinoma	2 Stage IV
			1 Carcinosarcoma	1 Stage IV
			1 Endometroid	1 Stage IV
			5 Serous	1 Stage Ia, 1 Stage IIIa, 1 Stage IIIb, 1 Stage IIIc, 1 Stage IV
**Other primary**^**a**^	1	Invasive epithelial	1 Serous	1 Stage IIIc
	1		1 Clear cell	1 Stage IIb
**(b) Cause of death (n = 40)**				
**Malignant neoplasm without specification of site (ICD-10 C80)**	27	Invasive epithelial	1 Adenocarcinoma	1 Stage IV
			1 Mucinous	1 Stage IV
			1 Carcinosarcoma	1 Stage IIIc
			10 Carcinoma	5 Stage IIIc; 5 Stage IV
			14 Serous	1 Stage Ic; 1 Stage IIIb; 8 Stage IIIc; 3 Stage IV;
**Multiple registrations which include malignant neoplasm without specification of site (ICD-10 C80) as well as nonovarian cancer**	10	Invasive epithelial	2 Carcinoma	1 Stage IIIc; 1 Stage IV
			5 Serous	4 Stage IIIc; 1 Stage IIIa
			1 Clear cell	1 Stage 2b
			1 Fallopian tube cancer (serous)	1 Stage IIIc
			1 Serous	1 Stage III
**Malignant neoplasm of ill defined,secondary and unspecified sites (abdomen) (icd-10 C76.2)**	1	Invasive epithelial	1 Carcinoma	1 Stage IIIc
**Carcinoma in situ of other and unspecified genital organs (ICD-10 D07.9)**	1		1 Serous	1 Stage IIIb
**Neoplasm of uncertain behavior of ovary (ICD-10 D39.1)**	1		1 Serous	1 Stage II b

^a^Malignant neoplasm of pelvis (ICD10 - C76.3); Malignant neoplasm of retroperitoneum (ICD10 - C48.0)

### Agreement of registry- and outcome review-assigned ovarian cancer deaths

At censorship, 2041 cases had undergone outcomes review and had a relevant ICD-10 death registration. Of the 656 ovarian cancer death registrations, 92.2% (605/656) were confirmed on outcomes review. The CoD in 51 (7.8%; 51/656) of 656 women registered as having died of ovarian, tubal or primary peritoneal cancer were reclassified on outcome review as non-ovarian. This includes malignant neoplasm of unknown origin (51.0%; 26/51), other malignancy (37.3%; 19/51) or non-cancer causes (11.8%; 6/51). Over half of the other cancer deaths were due to endometrial cancer (52.6%; 10/19) (Table 2). In addition, 40 (6.2%; 40/645) of 645 women confirmed by outcomes review as having died of tubo-ovarian cancer were registered as having died of non-tubo-ovarian cancer causes (Table 3). The agreement (86.3%; 1763/2041) between cancer registry and OR was substantial (kappa 0.78; 95% CI 0.76–0.80).

## Discussion

### Main findings

Our findings demonstrate that in trials using follow-up through national cancer registration alone in England, Wales and Northern Ireland, 15% of tubo-ovarian cancer cases may be missed. Similarly, the use of national death registration alone would result in 11% of tubo-ovarian cancer deaths being missed. The proportions of missing cancer registrations showed no decline over time unlike the deficit in disease-specific death registrations. These rates could be halved if additional national electronic datasets such as hospital episode statistics are used. When extrapolating these results, two facts must be taken into consideration. We included cancer and death registrations of primary peritoneal cancer in ovarian cancer incidence and mortality statistics. This is not the norm adopted by the UK Office of National Statistics or US SEER Registries. We obtained final follow-up data from the registries 3 months after the censorship date in order to fulfil reporting guidelines for the trial. Longer intervals from censorship to obtaining registry data are likely to reduce the rate in the last year.

Adjudication by an independent review process can improve the accuracy of cancer and death registrations. In those where a cancer registration was available, 9% of registry reported tubo-ovarian cancers would have inaccurate cancer site assignment. Additionally, 4% of the final outcomes confirmed cancers would have been missed as they were registered as non-ovarian cancers. Reliance on death registration alone would have resulted in a similar proportion (7.8%) of deaths being wrongly assigned to tubo-ovarian cancers and 6.2% of ovarian cancer deaths being missed.

### Strengths and limitations

A key strength of our analysis is that we compared cancer diagnoses and causes of death reported by the national registries of England, Wales and Northern Ireland with those assigned through independent central adjudication in a population cohort of over 3000 women. Of the 202,638 women, 202,632 (99.9%) women were electronically flagged using their NHS number to the relevant national cancer and death registries. The multiple additional data sources (Table 1) we used to identify potential ovarian cancer cases further ensured that we had a complete population dataset of cases.

We did not restrict the study to women with the tubo-ovarian cancer-specific ICD-10 codes (C56, C57.0, C48.1 and C48.2) but used 19 possible ICD-10 codes, in particular malignant neoplasm primary site unknown (ICD-10 C80) that might have potentially included tubo-ovarian cases.

The peritoneal cancers reported by cancer registries were included as tubo-ovarian in this analysis. While this is the norm among clinicians and researchers, these cancers are not included in national tubo-ovarian statistics. We did this to ensure that registry completeness rates were not underestimated due to the 2014 WHO revision of the primary site definition [8]. As the latter is adopted by pathology departments the world over, it is likely that cancers previously denoted as primary peritoneal will be registered as tubo-ovarian.

A limitation is that the cases were diagnosed over a prolonged period between 2001 and 2014. To address this, we explored time trends. While there were no time trends in missing cancer registration data, the completeness of death registration data improved over the years.

### Interpretation

The use of UK cancer registry data alone would have allowed us to detect 85% of the tubo-ovarian cancer cases. This is an improvement from the 78% sensitivity that we reported in our previous trial where women were diagnosed with these cancers between 1986 and 1993 [12]. It is likely that the rates have improved further in the more recent years. The use of multiple national electronic data sets especially hospital administration records can augment these rates. A recent report on tubo-ovarian cancers diagnosed between 2004 and 2012 in Switzerland also found that the hospital registry data provided complementary information to that from the cancer registries [13]. Sourcing data directly from live healthcare systems allows trials to overcome the latency in the national cancer registration systems. A median latency of 18 (range 4–60) months to completion of incidence ascertainment was reported in a recent survey of the European Network of Cancer Registries [14].

The overall agreement regarding ovarian cancer site between OR and cancer registries was moderate. A key area of discrepancy was related to tumours defined by the ICD-10 code D39.1, neoplasms of the ovary of unknown or uncertain behaviour. On outcome review, the majority were reclassified as malignant neoplasm of the ovary (ICD-10 codes C56), either borderline epithelial ovarian or granulosa cell tumours. Most of these discrepancies likely reflect historical coding practices and classification systems used by regional registries prior to centralization of services in 2013. Furthermore, coding for ovarian cancers since 2011 uses the ICD-O-3 system [15], where the topographical code (site of cancer) is clearly separated from the morphological code which includes the behaviour (malignant/benign). In the current system, as borderline ovarian tumours are classified as C56 (topographical code) with a morphology code of 1, there is less likelihood of the cases not being registered as ovarian cancer. However, our data for 2012 to 2014 are not sufficiently large to confirm this.

The 9% of tubo-ovarian cancers reported by the registries that were classed as ‘not ovarian’ cancers on review were equally distributed between three main groups: (1) miscoding of a benign mass as a cancer, (2) other primary cancer, and (3) malignant neoplasm, primary site unknown (C80). The other primary group included just under a third of cancers that metastasized to the ovary, which might have led to the confusion. The ovaries are a frequent site for metastases, with 5–30% of ‘ovarian masses’ being secondary metastasis reported from non-ovarian cancers [16].

The cause of death on death certificates is not always accurate [17] due to a variety of reasons (inexperience of certifying physician, lack of sufficient time/information, coding errors). However, in our trial, there was substantial agreement regarding the primary site between the death registry-reported ovarian cancer deaths and those assigned by the review committee. This has also been reported in screening trials of other cancers (e.g. prostate) which used adjudication panels [18, 19]. Nonetheless, 6.2% additional tubo-ovarian cancer deaths were identified through the adjudication process. A high proportion were registered as malignancies with an unknown primary site (C80) and all were at an advanced stage. In advanced cancer, where multiple sites are involved, it may be difficult to assign the primary site [20]. The lack of consistency in the approach adopted by pathologists to the assignment of the primary site during our trial led to a proposal for unified criteria for tubo-ovarian site assignment [21] which has since been adopted in international pathology reporting [22] and clinical guidelines [23].

The adjudication process used in the UKCTOCS trial is not dissimilar to that used in the Prostate Lung Colorectal and Ovarian Cancer screening trial [24]. In contrast to the cluster randomised trial of PSA testing for prostate cancer [25] where data was abstracted into vignettes for expert review, we provided copies of original clinical notes to the ORC members. To minimise ascertainment bias, any reference to the trial/randomisation arms was redacted in these documents. Review only occurred if the minimal required number of documents was available. ORC members who were highly experienced gynaecological oncology surgeons or pathologists used an algorithm developed specifically during the trial to assign a cancer site. To ensure accuracy and reproducibility over the years and across assessors, we audited the reviews and introduced a classification algorithm (Supplementary Figure 3, see Additional file 4). The resource implications of central adjudication can be significant, as it requires many hours of highly trained senior staff time. Often as in our trial, it is challenging to estimate costs as reviewers usually volunteer their time and work outside normal office hours. To save the effort and cost of adjudication, we used individual as opposed to group adjudication, a work flow that involved more than one assessment only when there was discrepancy between the primary reviewer and registered ICD-10 code, with group review limited to those where there was discrepancy between two reviewers.

Adjudication also provides information on other key variables such as staging and morphology which in the past have been poorly documented by registries [26] but now improving [27]. In our trial, we observed a stage shift in the multimodal screening arm [6]. This would not have been quantifiable using data from the registries alone without the detailed review of all available clinical documents as undertaken in UKCTOCS.

Kahan et al. [28] using a simulation model concluded that outcome misclassification can lead to biased treatment effect estimates and reduced power, potentially resulting in an erroneous conclusion regarding efficacy. The implementation of strategies to reduce misclassification is therefore of critical importance in clinical trials. In UKCTOCS, the main impact of adjudication was in improving the accuracy of tubo-ovarian cancer. On death outcomes, there was no significant difference on the mortality impact of screening between the analyses which used ORC-adjudicated disease-specific deaths and the sensitivity analysis limited to deaths with the disease-specific ICD-10 codes (ICD-10 C56, C57 and C48) for tubo-ovarian cancer [10].

## Conclusion

Our data suggests that follow-up of trial participants for tubo-ovarian cancer using national registry data will result in incomplete ascertainment particularly of the site due in part to the latency of registration. This can be reduced by using hospital administrative data, which has shorter latency. Central adjudication by experts though resource intensive adds value by improving the accuracy of diagnoses. Revised classification systems for ovarian cancer [8] will improve accurate identification and aid reproducibility of site assignment, but this will require pathologists and clinicians to apply this new system uniformly across the globe.

## Supplementary Information

**Additional file 1: Supplementary Table 1.** International Classifications of Disease, (Revision 10) used for data linkage with national cancer registries in England, Wales and Northern Ireland.

**Additional file 2: Supplementary Figure 1.** Outcomes Cancer Review Form.

**Additional file 3: Supplementary Figure 2.** Outcomes Death Review Form.

**Additional file 4: Supplementary Figure 3.** Algorithm for assignment of tubo-ovarian cancer site based on available data.

**Additional file 5: Supplementary Table 2.** Year of diagnosis / death of tubo-ovarian cancers with missing registration.

## Data Availability

The datasets used and/or analysed during the current study are available from the corresponding author on reasonable request.

## Abbreviations

UKCTOCS: United Kingdom Collaborative Trial of Ovarian Cancer Screening
ORC: Outcomes review committee
CoD: Cause of death
ROCA: Risk of Ovarian Cancer Algorithm
ONS: Office of National Statistics
BSO Northern Ireland: Business Services Organisation, Health and Social Care Northern Ireland
ICD10: International Classifications of Disease (Revision 10)
NHS: National Health Service
GP: General practitioners
iEOC: Invasive epithelial ovarian cancer
