# Supplementary material for: Completeness and accuracy of national cancer and death registration for outcome ascertainment in trials—an ovarian cancer exemplar
Source: Trials. 2021 Jan 25;22:88. doi: 10.1186/s13063-020-04968-x (PMC7831170; doi:10.1186/s13063-020-04968-x)
Supplement: Supplementary file 1 — Additional file 1: Supplementary Table 1. International Classifications of Disease, (Revision 10) used for data linkage with national cancer registries in England, Wales and Northern Ireland. [file 13063_2020_4968_MOESM1_ESM.pdf]

Table S1: International Classifications of Disease, (Revision 10) used for data linkage with national cancer registries in England, Wales and Northern Ireland

ICD10 code	Disease classification
C48.0	Malignant neoplasm of retroperitoneum
C48.1	Malignant neoplasm of specified parts of peritoneum: mesentery, mesocolon, omentum, peritoneum (parietal, pelvic)
C48.2	Malignant neoplasm of peritoneum, unspecified
C56	Malignant neoplasm of ovary
C57.0	Malignant neoplasm of Fallopian tube
C57.1	Malignant neoplasm of broad ligament
C57.4	Malignant neoplasm of uterine adnexa, unspecified
C57.7	Malignant neoplasm of other specified female genital organs
C57.8	Malignant neoplasm of overlapping lesion of female genital organs
C57.9	Malignant neoplasm of female genital organ, unspecified
C76.2	Malignant neoplasm of other abdomen
C76.3	Malignant neoplasm of pelvis
C80	Malignant neoplasm, primary site unknown, so stated
D07.3	Carcinoma in situ of other and unspecified female genital organs
D28.2	Benign neoplasm of uterine tubes and ligaments
D39.1	Neoplasm of uncertain or unknown behaviour of ovary
