# Supplementary material for: Completeness and accuracy of national cancer and death registration for outcome ascertainment in trials—an ovarian cancer exemplar
Source: Trials. 2021 Jan 25;22:88. doi: 10.1186/s13063-020-04968-x (PMC7831170; doi:10.1186/s13063-020-04968-x)
Supplement: Supplementary file 2 — Additional file 2: Supplementary Figure 1. Outcomes Cancer Review Form. [file 13063_2020_4968_MOESM2_ESM.pdf]

United Kingdom Collaborative Trial of Ovarian Cancer Screening

UKCTOCS

OUTCOMES CANCER REVIEW FORM

First Name ______________________ **Surname** ______________________________ **Volunteer Ref No.** __________________

Cancer **Yes / No**

Primary cancer site:

- ❑ Ovarian or Fallopian tube (C56/C57.0)
- ❑ Primary Peritoneal (C48)
- ❑ Malignant neoplasm, primary site unknown (C80)
- ❑ Malignant neoplasm, primary site unknown BUT NOT OV/FT (C80 but NOT OV/FT/PP)
- ❑ Breast
- ❑ Endometrium
- ❑ Other genital tract
- ❑ Other cancer– specified

Reviewed documentation:

- ❑ Death Certificate
- ❑ ONS Cancer Registration
- ❑ CA125 report
- ❑ Histology Report
- ❑ Cytology Report
- ❑ Histology review
- ❑ Surgery Notes
- ❑ MDT Meeting Summary
- ❑ Hospital Letter/Notes
- ❑ Discharge Summary
- ❑ Imaging Report
- ❑ Hospital Episode Statistics (HES)
- ❑ Other _________________________________

Histology of the ovary/fallopian tube:

- ❑ Primary invasive epithelial malignant neoplasm
- ❑ Primary borderline epithelial malignant neoplasm
- ❑ Primary non-epithelial malignant neoplasm
- ❑ Secondary malignant neoplasm
- ❑ Benign neoplasm
- ❑ Neoplasm of uncertain or unknown behaviour
- ❑ Normal adnexa
- ❑ Other (please describe in notes)
- ❑ No adnexal histology

- ❑ Second adnexal pathology present (details in notes)

Disease code (ICD 10) relating to primary cancer site:

Stage ____________________________

Grade ____________________________

Topography ____________________________

Morphology ____________________________

Additional morphology ______________________

Is this a type II cancer? ☐ Yes ☐ No ☐ Uncertain

Notes:

Synchronous/ Metachronous neoplasm (please circle)

Cancer site ____________________________

Morphology ____________________________

Stage ____________________________

Grade ____________________________

Unable to 'sign off':

- ❑ More information required
- ❑ Outcomes Committee Discussion
- ❑ Path Review

Signature : __________________________________ **Date:** ______/______/______

Outcomes Committee Form Version 3.8, September 2010
ISRCTN 22488978
