# Supplementary material for: Completeness and accuracy of national cancer and death registration for outcome ascertainment in trials—an ovarian cancer exemplar
Source: Trials. 2021 Jan 25;22:88. doi: 10.1186/s13063-020-04968-x (PMC7831170; doi:10.1186/s13063-020-04968-x)
Supplement: Supplementary file 3 — Additional file 3: Supplementary Figure 2. Outcomes Death Review Form. [file 13063_2020_4968_MOESM3_ESM.pdf]

United Kingdom Collaborative Trial of Ovarian Cancer Screening

OUTCOMES DEATH REVIEW FORM

First Name: **Surname:** **Volunteer Ref No.:**

**DC checked**	**Volunteer diagnosed with a cancer previously**	**Outcomes cancer form completed**
Yes / No	Yes / No	Yes / No

Cause of death

- ❑ Primary Ovarian or Fallopian tube
- ❑ Primary peritoneal
- ❑ Malignant neoplasm, primary site unknown (C80)
- ❑ Malignant neoplasm, primary site unknown BUT NOT OV/FT/PP (C80 but NOT OV/FT/PP)
- ❑ Other cancer – specified
- ❑ Other – non cancerous

If 'Other' give details and specify ICD-10 code:

If cause of death is OV, FT or PP progression based on (tick ALL applicable fields below):

- ❑ Appearance of new lesions on imaging
- ❑ Increase in the size of previously measured disease on imaging
- ❑ Clinical worsening of the symptoms and signs of disease in the absence of imaging
- ❑ Treatment complications

Confirmatory documentation for cause of death

- ❑ Documents checked on 'Outcomes Cancer Form'
- ❑ ONS Cancer Registration (related to cause of death)

Additional documents

- ❑ CA125 Report
- ❑ Radiology Report
- ❑ Histology Report
- ❑ Cytology Report
- ❑ Surgery Notes
- ❑ MDT Summary
- ❑ Hospital Letter/Notes
- ❑ Hospital Discharge Summary
- ❑ Hospice Discharge Summary
- ❑ Hospital Episode Statistics (HES)
- ❑ Other ________________________________

Unable to 'sign off'

- ❑ More information required
- ❑ Complex case –Outcomes Committee Discussion
- ❑ Other - specify

Notes:

Signature : ______________________________ **Date:** _____/_____/_____
