# Supplementary material for: Completeness and accuracy of national cancer and death registration for outcome ascertainment in trials—an ovarian cancer exemplar
Source: Trials. 2021 Jan 25;22:88. doi: 10.1186/s13063-020-04968-x (PMC7831170; doi:10.1186/s13063-020-04968-x)
Supplement: Supplementary file 4 — Additional file 4: Supplementary Figure 3. Algorithm for assignment of tubo-ovarian cancer site based on available data. [file 13063_2020_4968_MOESM4_ESM.pdf]

Documentation available:		Primary cancer site
Histology definite of ovarian or fallopian tube origin	☐	**C56/C57.0**
Histology definite for PPC (**ADNEXA NORMAL OR SHOWING ONLY SECONDARY INVOLVEMENT)**	☐	**C48**
Histology suggestive of other primary **(TICK ONLY IF PRIMARY SITE IS SPECIFIED)**	☐	**Other primary**

**Clinical details**	**CA125**	**Adnexal imaging**		**Other Imaging**		**Cytology (ascites)**	**Pathology**		
Clinical history and/or findings in keeping with OV/FT/PP malignancy	CA125 elevated	Adnexal mass	Normal-sized adnexae	Peritoneal/omental disease in keeping with OV/FT/PP malignancy	Imaging suggests non-OV/FT/PP malignancy	Adenocarcinoma cells	Pathology (any site) compatible with OV/FT/PP malignancy	Pathology (any site) indicates/confirms non-OV/FT/PP malignancy	**Primary cancer site**
☐	☐	☐	☐	☐	☐	☐	☐	☐	
√		√					√		**C56/C57**
√		√				√			
√		√		√					
√	√			√			√		
√	√			√		√			
√	√		√	√			√		**C56/C57/C48**
√	√		√	√		√			
√	√		√		√		√		
		√			√				**C80 but NOT OV/FT/PP**
			√		√				
					√				
	√							√	
√						√ (Malignant cells)			**C80**

(pink) **Irrespective of whether this column is positive or negative**
